# Hematological parameters and all-cause mortality: a prospective study of older people

**DOI:** 10.1007/s40520-017-0791-y

**Published:** 2017-06-29

**Authors:** Joanna Frąckiewicz, Dariusz Włodarek, Anna Brzozowska, Elżbieta Wierzbicka, Małgorzata Anna Słowińska, Lidia Wądołowska, Joanna Kałuża

**Affiliations:** 1 Department of Human Nutrition, Warsaw University of Life Sciences-SGGW, Warsaw, Poland; 2 Department of Dietetics, Warsaw University of Life Sciences-SGGW, Warsaw, Poland; 3 Community Health Center Warszawa Bemowo-Włochy, Warsaw, Poland; 4 Department of Human Nutrition, University of Warmia and Mazury in Olsztyn, Olsztyn, Poland

**Keywords:** Mortality, Older people, Hematological parameters, Prospective study, Gender

## Abstract

**Background:**

The effect of low and high concentration of some hematological parameters in the blood can have a negative impact on health.

**Aim:**

Therefore, we investigated the associations between hematological parameters and all-cause mortality among older people living in Poland.

**Methods:**

The study was carried out among 75–80-year-old participants (*n* = 403) from Warsaw and Olsztyn regions, Poland. Information on lifestyle factors and food consumption were obtained at baseline (June 1, 1999) using a self-administered questionnaire. Red blood cell, haemoglobin, hematocrit, mean corpuscular volume (MCV), mean corpuscular haemoglobin (MCH), and mean corpuscular haemoglobin concentration (MCHC) were determined. The data on deaths from all-causes were collected from the baseline until October 31, 2006. During an average of 7.4 years of follow-up, we ascertained 154 cases of death from all-causes.

**Results:**

Compared with men in the lowest tertile of MCV, MCH, and MCHC, the multivariable hazard ratios (HRs) of all-cause mortality in those in the highest tertile were 0.35 (95% CI, 0.17–0.73), 0.32 (95% CI, 0.16–0.67), and 0.44 (95% CI, 0.22–0.88), respectively. In contrast, among women after combining the second and the third tertiles of MCV, MCH, and MCHC, the HRs were 2.01 (95% CI, 1.01–3.99), 1.71 (95% CI, 0.85–3.43), and 1.09 (95% CI, 0.62–1.94), respectively.

**Discussion/conclusion:**

We observed inverse associations between some hematological parameters and all-cause mortality among men, but not among women. This may be explained by a difference in iron metabolism, iron status, hormone regulations, or the occurrence of some diseases.

## Introduction

The prevalence of anaemia increases dramatically with advancing age, reaching nearly 50% in older people, and will be increased further due to population aging [1–3]. Most anaemia cases in older individuals result from iron deficiency, chronic inflammation, chronic kidney disease, or some of them may be unexplained [4–6]. Anaemia of inflammation is one of the main types of anaemia in the geriatric population and is connected to many age-related diseases such as: obesity, cancer, chronic renal disease, etc. Even mild anaemia is related to increased mortality [7, 8]. Low haemoglobin (Hb) concentration contributes to pathological conditions, such as poor functional status, cognitive decline and dementia, increased risk of hospitalization, morbidity, and mortality [9–12].

In contrast, a few studies have shown that a high red blood cell (RBC) count, even within the normal reference range, was strongly associated with an increased risk of all-cause mortality and cardiovascular (CVD) incidence and mortality in middle-aged and elderly people [13–17]. The Framingham Heart Study indicated that older men with hematocrit (HTC) above 48% compared to those with HTC 45–46% had a statistically significantly higher risk of CVD and a tendency to higher risk of coronary heart mortality [18].

Until now, most studies determined the associations between Hb and HTC and the risk of mortality among elderly, but did not test other hematological parameters, such as mean corpuscular volume (MCV), mean corpuscular haemoglobin (MCH) and mean corpuscular haemoglobin concentration (MCHC).

Given the inconsistent results of the studies on Hb and HTC and lack of studies on MCV, MCH, and MCHC and risk of morbidity and mortality among the older people, we investigated the associations of hematological parameters commonly tested by General Practitioners (GPs) with all-cause mortality in 75–80-year-old men and women living in two districts of Poland.

## Methods

### Study design and population

**Fig. 1:**
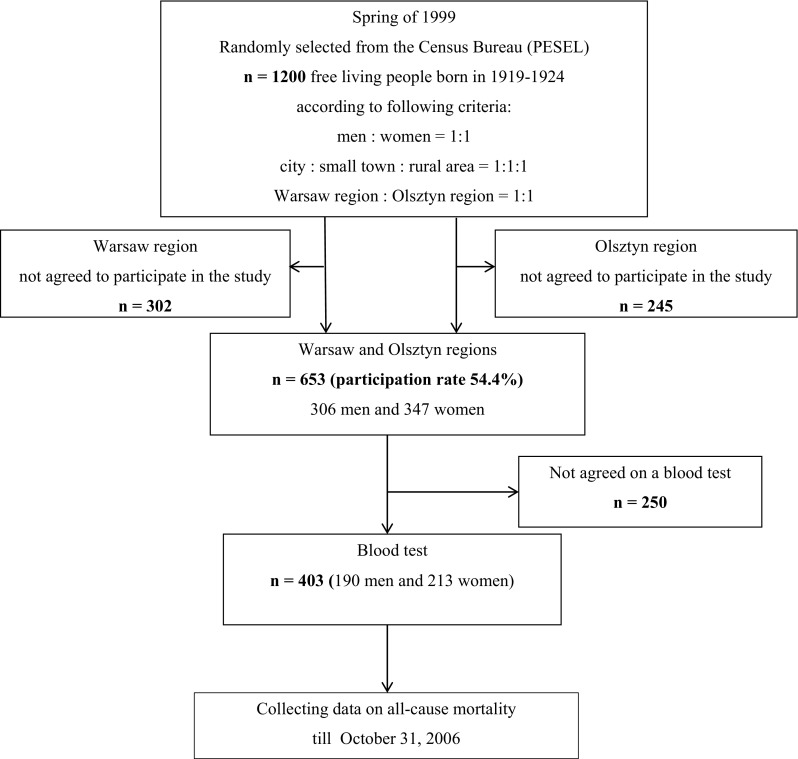
Flow diagram of recruitment and participation in the study

In the spring of 1999, 1200 free living respondents aged 75–80 years (born in 1914–1924) were randomly selected from the Census Bureau according to the Personal Identification Number (PESEL) by quota 600 participants from the Warsaw region and 600 from the Olsztyn region, Poland (Fig. 1). In each region, the proportion of selected men and women was 1:1 and the proportion of people living in cities, small towns, and rural areas was 1:1:1. The participation rate in the study was 54.4% (306 men and 347 women). Among these participants, 403 people (190 men and 213 women) have agreed to a blood test and those people were included in the analysis. The study was approved by the Regional Ethics Commission located at the National Food and Nutrition Institute, Warsaw, Poland.

### Assessment of diet and other exposure

In the spring of 1999, after a pilot study carried out among 60 participants from the Warsaw region and 60 participants from the Olsztyn region, the main study was conducted. Data on food consumption were collected using 3-day records in Warsaw and 24-h recalls in the Olsztyn region. Information on physical activity, education, smoking status, avoiding alcohol, dietary supplement use, and medicine use was obtained at baseline using a self-administered questionnaire. The body mass index (BMI) was calculated by dividing the weight (kg) by the square of height (m).

### Hematological parameters and total serum cholesterol level

Blood parameters were determined in the medical laboratory of the National Food and Nutrition Institute in Warsaw and in the medical laboratory of the Municipal Polyclinic Hospital in Olsztyn. RBC, Hb, HCT, MCV, MCH, and MCHC in blood were determined using the Biocode Hycel Celly Hematology Analyzer (Biocode Hycel, Rennes, France) in Warsaw and the Minos analyzer (ABX, France) in Olsztyn. Total cholesterol was measured in serum using the Vitros 250 analyzer (Johnson & Johnson Clinical Diagnostic, New Zealand) in Warsaw and the Cobas Mira E analyzer (La Roche, Switzerland) in Olsztyn.

### Case ascertainment

The data on deaths from all causes were collected from June 1, 1999 until October 31, 2006. The data on deaths were obtained from the Register Offices individually for each participant.

### Statistical analysis

Cox proportional hazards regression models were used to estimate hazard ratios (HRs) and 95% confidence intervals (CIs) of all-cause mortality. We categorized participants into tertiles of RBC, Hb, HCT, MCV, MCH, and MCHC, separately for men and women. Among women in the result description, the second and third quartiles of hematological parameters were combined because of a relatively small number of deaths and unstable HRs.

The multivariable models included the following variables: age (continuous variable), region (Warsaw or Olsztyn), physical activity (very low and low, or moderate and high), education (lower then higher, or higher and university), smoking status (yes or no), avoiding alcohol (yes or no), supplement use (yes or no), medicine use (yes or no), body mass index (≤25 or >25 kg/m^2^), serum cholesterol level (tertiles), and intake of energy and iron (tertiles). The multivariable models were calculated separately for each tested hematological parameter.

To compare survival curves, the Kaplan–Meier method and the log-rank test were used. The proportional hazards’ assumption was evaluated by regressing scaled Schoenfeld residuals against survival time. There was no evidence of departure from the assumption. To calculate *p* values for trend, the median values of tertiles for RBC, Hb, HCT, RBC, MCV, MCH, and MCHC were used as a continuous variable. Using the likelihood ratio test, we tested statistical interactions between tested hematological parameters in predicting all-cause mortality according to sex, physical activity, BMI, smoking status, and avoiding alcohol.

The statistical analyses were performed using Statistica version 10.0 PL and IBM SPSS Statistics version 21. The Shapiro–Wilk test was used to check the data distribution. Average values of the tested parameters were compared using the Mann–Whitney *U* test, while categorical data were analyzed using the Chi^2^ Pearson test. All reported *p* values were two-sided, and *p* values ≤0.05 were considered as statistically significant.

## Results

The mean age of participants was 77.0 ± 1.7 years (Table 1). Over half of the respondents were women (52.9%) and lived in the Warsaw region (53.3%). Most participants evaluated their physical activity as moderate or high and had primary or secondary education. Twenty percent of men and about six percent of women were current smokers, and 14 and 22%, respectively, declared to avoid alcohol. In the past 12 months, dietary supplements and medicine were used by 41 and 72% of participants, respectively. About 73% of respondents had a BMI higher than 25 kg/m^2^. The concentration of serum cholesterol was statistically significantly higher in women compared to men, while the intake of energy and iron was significantly lower in women.

**Table 1:** Baseline characteristic of the study population (% of the respondents)

Variables	Total *N* = 403	Men *N* = 190	Women *N* = 213	Difference between men and women *p* value
Age (years)^a^	77.0 ± 1.7^a^	77.2 ± 1.7	76.9 ± 1.6	0.071^b^
Region				
Warsaw	53.2	53.2	53.3	0.301^c^
Olsztyn	46.8	46.8	46.7	
Self-reported physical activity				
Very low or low	29.5	25.3	33.2	0.082^c^
Moderate or high	70.5	74.7	66.8	
Education				
Primary or secondary	72.5	68.4	76.2	0.082^c^
Higher	27.5	31.6	23.8	
Current smoker	12.4	20.0	5.6	<0.001^c^
Avoiding alcohol	18.3	14.2	22.0	0.044^c^
Dietary supplement use	40.6	31.3	49.1	<0.001^c^
Medicine use in the past 12 months	72.0	66.8	76.6	0.023^c^
Body mass index (kg/m^2^)^a^	28.6 ± 5.2	27.4 ± 4.0	29.6 ± 5.9	0.028^b^
≤25	26.8	32.1	21.5	0.066^c^
>25	73.2	67.9	78.5	
Serum cholesterol (mmol/l)^a^	5.83 ± 1.13	5.54 ± 1.10	6.08 ± 1.09	<0.001^b^
Intake of energy (kcal/d)^a^	1635 ± 650	1902 ± 691	1398 ± 505	<0.001^b^
Intake of iron (mg/d)^a^	9.06 ± 5.23	10.7 ± 5.44	7.62 ± 4.58	<0.001^b^

^a^Mean ± SD (standard deviation)

^b^U-Mann–Whitney test

^c^χ^2^ Pearson test

RBC and concentration of Hb, HCT, MCH, and MCHC were significantly higher among men than they were among women (Table 2). We observed a higher percentage of men compared to women with the following parameters below reference values: RBC (43.1 vs. 15.5%, respectively), Hb (12.6 vs. 1.4%, respectively), and HCT (27.5 vs. 16.0%, respectively). At the same time, the percentage of men with MCV (21.7%) and MCH (24.5%) above reference values was higher in comparison to women (15.1% and 13.5%, respectively).

**Table 2:** Hematological parameters among 75–80-year-old participants

Hematological parameters	Total	Men	Women	Difference between men and women *p* value
RBC (10^6^/µl)				
Mean ± SD	4.46 ± 0.49	4.56 ± 0.56	4.38 ± 0.39	<0.001^b^
Minimum–maximum	2.19–7.45	2.19–7.45	3.19–5.79	
Number of participants (%)				
<Reference	28.4	43.1	15.5	<0.001^c^
Reference^a^	70.6	55.9	83.6	
>Reference	1.0	1.0	0.9	
Hb (g/dl)				
Mean ± SD	13.5 ± 1.4	14.0 ± 1.5	13.1 ± 1.1	<0.001^b^
Minimum–maximum	9.0–18.9	9.0–18.9	10.7–17.7	
Number of participants (%)				
<Reference	6.7	12.6	1.4	<0.001^c^
Reference^a^	91.3	84.8	97.2	
>Reference	2.0	2.6	1.4	
HCT (%)				
Mean ± SD	40.8 ± 3.8	41.9 ± 4.4	39.7 ± 2.8	<0.001^b^
Minimum–maximum	25.7–57.9	25.7–57.9	33.1–49.4	
Number of participants (%)				
<Reference	21.5	27.5	16.0	0.011^c^
Reference^a^	77.6	70.9	83.5	
>Reference	0.9	1.6	0.5	
MCV (fl)				
Mean ± SD	91.6 ± 6.2	92.4 ± 6.4	90.0 ± 5.9	0.065^b^
Minimum–maximum	74.0–120.0	74.0–117.0	76.9–120.0	
Number of participants (%)				
<Reference	0.5	1.1	0.0	0.069^c^
Reference^a^	81.3	77.2	84.9	
>Reference	18.2	21.7	15.1	
MCH (pg)				
Mean ± SD	30.4 ± 2.2	30.8 ± 2.3	30.0 ± 2.0	0.002^b^
Minimum–maximum	21.5–40.9	21.5–40.9	24.6–39.7	
Number of participants (%)				
<Reference	3.8	3.3	4.3	0.024^c^
Reference^a^	77.6	72.2	82.2	
>Reference	18.6	24.5	13.5	
MCHC (g/dl)				
Mean ± SD	33.2 ± 1.1	33.4 ± 1.2	33.0 ± 1.0	0.016^b^
Minimum–maximum	29.1–36.8	29.1–36.8	30.2–36.1	
Number of participants (%)				
<Reference	8.9	8.1	9.7	0.593^c^
Reference^a^	91.1	91.9	90.3	
>Reference	0.0	0.0	0.0	

*RBC* red blood cell, *Hb* haemoglobin, *HCT* hematocrit, *MCV* mean corpuscular volume, *MCH* mean corpuscular haemoglobin, *MCHC* mean corpuscular haemoglobin concentration

^a^Reference values: RBC 4.5–6.5 × 10^6^/µl in men and 4.0-5.5 × 10^6^/µl in women, Hb 12.5–17.0 g/dl in men and 11.2–15.5 g/dl in women, HCT 40–54% in men and 37–47% in women, MCV 76–96 fl in men and women, MCH 27–32 pg in men and women, MCHC 32–38 g/dl in men and women, according to laboratory reference limits

^b^U-Mann–Whitney test

^c^χ^2^ Pearson test

During an average of 7.4 years (3784 person-years, 1999–2006) of follow-up, we ascertained 154 cases of deaths from all-causes, including 81 deaths in men (42.6%) and 73 deaths in women (34.3%). This difference between men and women was not significant (*p* = 0.08).

We observed statistically significant interactions between hematological parameters (MCV and MCH) and sex in relation to all-cause mortality (*p* for interaction: 0.004 and 0.005, respectively); therefore, all analysis were conducted separately for men and women.

**Fig. 2:**
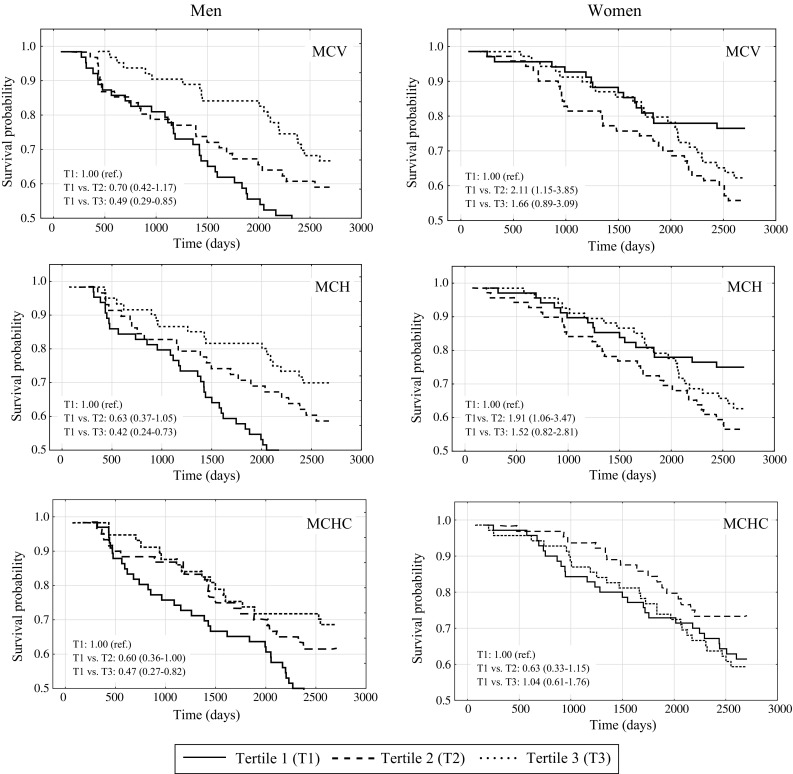
Survival curves for MCV, MCH, and MCHC among men and women. *Solid line* Tertile 1 (T1), *dashed line* Tertile 2 (T2), *dotted line* Tertile 3 (T3)

After adjusted HRs for possible confounders (multivariable HRs), we observed statistically significant inverse associations of some hematological parameters with risk of all-cause mortality among men (Table 3). Compared with men in the lowest tertile of MCV, MCH, and MCHC, the HRs of all-cause mortality in those in the highest tertile were 0.35 (95% CI, 0.17–0.73), 0.32 (95% CI, 0.16–0.67) and 0.44 (95% CI, 0.22–0.88), respectively. Among women in the second tertile versus those in the first tertile, the HR of all-cause mortality was 2.43 (95% CI, 1.20–4.92) for MCV and 2.12 (95% CI, 0.99–4.50) for MCH, while among women in the third tertile, the HRs were 1.44 (95% CI, 0.65–3.19%) and 1.40 (95% CI, 0.66–2.99), respectively. After combining the second and the third tertiles of MCV, MCH, and MCHC in women, the HRs were 2.01 (95% CI, 1.01–3.99), 1.71 (95% CI, 0.85–3.43), and 1.09 (95% CI, 0.62–1.94), respectively. Survival curves by tertiles of MCV, MCH, and MCHC in the group of men and women were presented in Fig. 2. We did not observe statistically significant associations by tertiles of RBC, Hb, and HCT with all-cause mortality among men and women.

**Table 3:** Tertiles of hematological parameters in participants and Hazard Ratios (95% confidence Intervals) of all-cause mortality

	Men				Women			
	Tertile 1	Tertile 2	Tertile 3	*p* for trend	Tertile 1	Tertile 2	Tertile 3	*p* for trend
RBC 10^6^/µl (median)	≤4.34 (4.05)	4.35–4.75 (4.60)	>4.76 (4.98)		≤4.24 (4.06)	4.25–4.52 (4.38)	>4.53 (4.74)	
No. of subjects	63	63	62		73	69	71	
No. of cases	25	26	30		27	27	19	
Age-adjusted HR	1.00	1.11(0.64–1.92)	1.30 (0.76–2.23)	0.340	1.00	1.12 (0.66–1.91)	0.73 (0.41–1.32)	0.108
Multivariable HR^a^	1.00	1.54 (0.75–3.18)	1.38 (0.65–2.91)	0.550	1.00	1.21 (0.65–2.25)	0.70 (0.36–1.36)	0.208
Hb g/dl (median)	≤13.5 (12.8)	13.6–14.5 (14.0)	>14. (15.3)		≤12.6 (12.1)	12.7–13.5 (13.1)	>13.6 (14.2)	
No. of subjects	70	59	61		71	81	61	
No. of cases	36	24	21		27	26	20	
Age-adjusted HR	1.00	0.75 (0.44–1.26)	0.59 (0.34–1.01)	0.105	1.00	0.90 (0.52–1.54)	0.94 (0.52–1.68)	0.999
Multivariable HR^a^	1.00	0.82 (0.42–1.60)	0.68 (0.35–1.33)	0.141	1.00	1.03 (0.54–1.96)	0.93 (0.49–1.78)	0.871
HCT % (median)	≤40.5 (38.4)	40.6–43.3 (41.9)	>43.4 (45.5)		≤38.5 (37.1)	38.6–40.7 (39.5)	>40.8 (42.4)	
No. of subjects	65	62	62		72	70	70	
No. of cases	32	23	26		27	24	22	
Age-adjusted HR	1.00	0.72 (0.42–1.24)	0.77 (0.46–1.29)	0.500	1.00	0.92 (0.53–1.60)	0.89 (0.51–1.56)	0.984
Multivariable HR^a^	1.00	0.86 (0.43–1.72)	0.77 (0.38–1.56)	0.436	1.00	0.90 (0.47–1.73)	0.76 (0.39–1.45)	0.636
MCV fl (median)	≤89.7 (87.0)	89.8–94.8 (91.6)	>94.9 (98.0)		≤88.0 (86.3)	88.1–92.0 (90.3)	>92.1 (96.0)	
No. of subjects	64	62	63		71	71	70	
No. of cases	34	25	21		16	31	26	
Age-adjusted HR	1.00	0.69 (0.41–1.15)	0.49 (0.29–0.85)	0.005	1.00	1.99 (1.08–3.64)	1.64 (0.88–3.06)	0.007
Multivariable HR^a^	1.00	0.66 (0.35–1.21)	0.35 (0.17–0.73)	0.010	1.00	2.43 (1.20–4.92)	1.44 (0.65–3.19)	0.117
MCH pg (median)	≤30.0 (29.1)	30.1–31.5 (30.8)	>31.6 (32.5)		≤29.3 (28.9)	29.4–30.7 (30.0)	>30.8 (31.8)	
No. of subjects	65	58	61		71	69	68	
No. of cases	37	24	18		17	30	25	
Age-adjusted HR	1.00	0.64 (0.39–1.08)	0.43 (0.24–0.75)	< 0.001	1.00	1.82 (1.00–3.31)	1.55 (0.84–2.87)	0.026
Multivariable HR^a^	1.00	0.67 (0.34–1.31)	0.32 (0.16–0.67)	0.001	1.00	2.12 (0.99–4.50)	1.40 (0.66–2.99)	0.139
MCHC g/dl(median)	≤32.8 (32.3)	32.9–33.7 (33.3)	>33.8 (34.6)		≤32.5 (32.1)	32.6–33.2 (32.9)	>33.3 (34.0)	
No. of subjects	66	60	59		73	65	69	
No. of cases	38	23	18		27	17	28	
Age-adjusted HR	1.00	0.58 (0.35–0.98)	0.46 (0.26–0.80)	0.010	1.00	0.60 (0.33–1.10)	1.00 (0.59–1.70)	0.854
Multivariable HR^a^	1.00	0.63 (0.34–1.18)	0.44 (0.22–0.88)	0.032	1.00	0.82 (0.42–1.61)	1.60 (0.81–3.17)	0.398

*RBC* red blood cell, *Hb* haemoglobin, *HCT* hematocrit, *MCV* mean corpuscular volume, *MCH* mean corpuscular haemoglobin, *MCHC* mean corpuscular haemoglobin concentration

^a^Multivariable model adjusted for age (continuous variable), region (Warsaw or Olsztyn), physical activity (very low and low, or moderate and high), education (primary or secondary, or higher), smoking status (yes or no), avoiding alcohol (yes or no), supplement use (yes or no), medicine use (yes or no), body mass index (≤25 or >25 kg/m^2^), serum cholesterol level (tertiles), and intake of energy and iron (tertiles)

Moreover, there were no statistically significant interactions between the tested hematological parameters and the smoking status, physical activity, alcohol consumption, and BMI (all *p*-interaction > 0.1).

## Discussion

In this study of older people, some hematological parameters were inversely associated with a risk of all-cause mortality among men, but not among women. Men in the highest versus those in the lowest tertile of MCV, MCH, and MCHC had a statistically significant 65, 68, and 56% lower risk of all-cause mortality, respectively. In contrast, women in the higher tertiles of MCV (the second and the third tertiles combined) in comparison to those in the first tertile had a twofold higher risk of all-cause mortality. No similar associations were observed for RBC, Hb, and HCT. Moreover, in our previous study in the same population of men and women, we observed that women with iron serum concentration above the median had a statistically significantly fourfold (HR: 4.10; 95% CI: 1.16–14.5) higher risk of all-cause mortality than those with lower iron serum concentration. In men, a similar association was not observed [19].

To the best of our knowledge, there are no previously published studies on MCV, MCH, and MCHC in relation to all-cause and specific-cause mortality. Most epidemiological studies have investigated the association between Hb [9, 20–23] and HCT [24–26] and the risk of all-cause mortality [9, 20–23] and CVD mortality [23–26] among people aged 55 years and older. The HRs of all-cause mortality increased from 33% in men and women with low Hb concentration to twofold in people with mild anaemia and sixfold in those with moderate and severe anaemia [23]. Results of a large prospective cohort study (*n* = 49,983) indicate a U-shape relationship between HCT and all-cause and CVD mortality in women; low and high values of HCT were associated with higher risk of death [24]. In addition, in a case–control study HCT ≥ 50% resulted in an 80% higher risk of coronary heart disease (CHD) mortality in men [22]. No similar associations between HCT and CHD were observed in the Second National Health and Nutrition Examination Survey [26].

In our study, we did not find any significant associations between Hb and HCT and all-cause mortality among men and women. However, among men, the HRs were 32 and 23% lower in the third tertile compared to the first tertile of Hb and HCT, respectively, while among women, the HR was 24% lower only for HCT. These results are consistent with the percentage of people below reference levels for these parameters. We suppose that the lack of a significant impact of Hb on all-cause mortality was a result of a small number of anemic women in our study (1.4% of women) and a relatively low percentage of anemic men (12.6%).

We can speculate that the gender differences in the presented results can depend on different iron metabolism and iron status as well as different hormone regulations and health condition. It is known that androgen and estrogen concentrations may play an important role in erythro- and myelopoiesis processes [27]. Based on a clinical trial, it was observed that a testosterone-induced increase in Hb and HCT was associated with stimulation of erythropoietin and reduced ferritin and hepcidin concentrations among elderly men [28]. Moreover, high risk of all-cause mortality among women may be caused by a potential adverse effect of iron. It is known that an excess of iron has pro-inflammatory and pro-oxidative properties [29, 30]. By catalysing the formation of free radicals, high levels of iron may lead to lipid peroxidation [31–33], protein, and DNA modifications [33, 34]. It was observed that MCH and MCHC were inversely correlated with C-reactive protein [35], and MCHC was associated with carotid intima media thickness and with interleukin-6 (IL-6) among hypertensive patients [36]. It seems feasible that the observed lower mortality in men in the highest versus those in the lowest tertile of MCV may be related to the level of red cell distribution width (RDW). It has been shown that the level of RDW inversely correlated with MCV [15, 37] and higher RDW is a strong predictor of all-cause mortality in adults of 45 and older [15, 38] as well as in older people with and without age-associated diseases [38].

The major strengths of the present study were the broad use of hematology parameters, a random selection of people from two different districts of Poland as well as data collection in both regions based on the same questionnaires. We adjusted the Cox proportional hazard models for the main potential confounders, which could affect the final results; however, we were not able to rule out residual or unmeasured confounding. The main limitation of this study was a relatively small number of participants and instances of death from all-causes. People who refused to participate in the study were characterized by a worse health status than those who took part; thus, the study population was healthier than the older Polish public. Another limitation of the study was the lack of data on the ferritin, transferrin receptor concentrations, and red cell distribution width as well as inflammation markers. Parameters such ferritin and transferrin receptor concentrations are more specific to determine iron status; however, hematological parameters are commonly examined in older people by GPs and they are useful prognostic factors of morbidity and mortality.

## Conclusions

The findings from this prospective study indicate that some hematological parameters were inversely associated with the risk of all-cause mortality among older men, but not among women. The differences in the results between genders can be explained by different iron metabolism, iron status, and hormone regulations among men and women, and also by the occurrence of diseases, which can have an impact on hematological parameters. This finding warrants confirmation in further prospective studies conducted on a bigger population.
